# Hybrid Ceramics Cementation Protocols: Scope Review

**DOI:** 10.4317/jced.61733

**Published:** 2024-09-01

**Authors:** Ícaro Menezes Beltrami, Cássia Cunha de Lima, Camila Teixeira do Nascimento, José Guilherme Pereira Gonçalves, Josuel Siqueira Azarias, Ana Laura Ferreira Bortoleto, Marcelo Coelho Goiato, Daniela Micheline dos Santos

**Affiliations:** 1 São Paulo State University (UNESP), School of Dentistry, Araçatuba, Brazil

## Abstract

**Background:**

Compared to high modulus repair materials such as zirconia and alumina, hybrid polymer-ceramic materials have lower stress concentrations in the tooth structure and bonding interface. The aim of this study is to evaluate viable cementation protocols through an integrative review and meta-analysis.

**Material and Methods:**

This systematic review was based on the PRISMA criteria. An electronic search was carried out in seven databases: Web of Science, Embase, PubMed, Clinical Trials, Scopus, Cochrane, Periodicals of Capes. The following inclusion criteria were used: hybrid ceramics, surface treatment protocol and which union test was presented, discarding duplicate articles and studies that did not meet the inclusion criteria. The articles were analyzed and selected through the RayYan platform.

**Results:**

Of the 160 articles identified, 24 studies were included in this review. The materials used by the authors were the hybrid ceramics: Vita ENAMIC, LAVA ULTIMATE, Cerasmart, Shofu Block, and the possible surface treatment protocols presented involving the use of: HF, Alumina blasting with or without silica, Silane, Laser and adhesive. The tests were carried out at different times and concentrations of the analyzed materials.

**Conclusions:**

With the studies that were included in this review, it was possible to show that the corrosion made by HF continues to be the gold standard for the treatment of surfaces in hybrid ceramics.

** Key words:**Hybrid ceramic, Bond Strength, Cementation.

## Introduction

Known for their excellent ability to artificially reproduce natural teeth, dental ceramics were introduced in dental applications due to their physical and mechanical characteristics, in addition to their optical qualities (1). Clinically, ceramics stand out among restorative materials for presenting excellent properties such as translucency, chemical stability, durability and biocompatibility (2).

Dental ceramics can be classified into (1): vitreous ceramics, which have a lower amount of mineral load, compared to their vitreous phase, responsible for viscosity and thermal expansion, thus providing greater aesthetics and translucency; polycrystalline ceramics, which have a high amount of crystalline phase, giving them better mechanical properties such as high compressive strength compared to vitreous ones; hybrid or resin matrix ceramics, which were developed with the purpose of providing adequate hardness to withstand chewing forces associated with satisfactory aesthetics, allowing for better stress distribution (3).

Hybrid ceramics, compared to vitreous and polycrystalline ceramics, have a lower degree of hardness and modulus of elasticity, providing low abrasive potential to the opposite dentition (4), which makes the use of this material very attractive. Hybrid ceramics are composed of fillers of zirconia and silica (5) nanoceramic particles associated with organic resin particles and polymers, providing resistance and aesthetics to the material. Regarding their manufacturing, they are made using the digital system Computer-aided design/Computer-aided manufacturing (CAD/CAM), with the main advantage being the precise adaptation of the restoration (4) to the tooth structure, as well as the speed of its manufacture.

The adhesion of hybrid ceramics to the tooth structure depends on adhesive cementation to provide better retention and longevity. These restorations will be exposed to chemical, thermal and mechanical challenges in the oral cavity, which may cause weakening of the adhesive interface4, with the possibility of the restoration loosening over time. Therefore, it is essential to treat the internal surface of the prosthetic restoration, creating microporosities on its surface with the aim of expanding the contact area with the resin cement (3).

There are different resources to optimize the interaction of resin cement with hybrid ceramics. It is known that the use of hydrofluoric acid in concentrations of 5% or 10% is the surface treatment of choice for glass ceramics (3), as it modifies the microstructure of the ceramic, creating microporosities, therefore being reliable and predicTable 6. Furthermore, there are other protocols for this purpose, such as air particle abrasion, aluminum oxide blasting and laser conditioning (4), all aiming to improve the adhesion capacity of the ceramic restoration to the resin cement used and the tooth structure.

However, little is the discussed in the literature about the surface treatment that should be carried out on restorations made with hybrid ceramics, with dental surgeons using surface treatments similar to vitreous and polycrystalline ceramics.

The objective of this research was to evaluate the different cementation protocols for hybrid ceramics, through an integrative review of scientific databases, presenting the surface treatment effects of each method found, highlighting the most favorable ones to obtain a better cement union. resinous to hybrid ceramics and tooth structure.

## Material and Methods

To clarify concepts and identify cementation protocols in hybrid ceramics, the method chosen for this work was the scoping review.

This type of review aims to identify gaps in the existing literature, achieving in-depth and broad results, which may or may not lead to a subsequent complete systematic review. A scoping study is conducted in the following steps: identification of the research question; identification of relevant studies; study selection; data mapping and gathering, summary and reporting of results.

-Step 1 - Identification of the research question

The PCC strategy (acronym for P: population; C: concept; C: context) was used, in accordance with PRISMA Extension for Scoping Reviews (PRISMA-ScR), published by the Joanna Briggs Institute (JBI). Thus, in this work, population (Hybrid Ceramics), concept (Surface treatment ) and context (Efficacy of each treatment performed were defined and the guiding question for study was then identified: “What is the most suiTable surface treatment for cementing hybrid ceramics?”.

-Step 2 - Identification of relevant studies

To carry out the search, the databases Web of Science, Embase, PubMed, Clinical Trials, Scopus, Cochrane and Periódicos da Capes were chosen. To survey the articles, the descriptors “Hybrid ceramic” = “Cad/Cam Polymer”, “Bond Strength” = “Cad/Cam restoration Prosthetic restoration”, “Cementation” = “Adhesive Cementation” and synonyms of these descriptors were applied according to Medical Subject Headings (MeSH). To complete the search strategy used in the databases, we applied the Boolean operators “ OR “ between the descriptors and their synonyms and “ AND “ between the descriptors referring to cementation protocols in hybrid ceramics. 160 relevant studies were identified in databases and gray literature, which were archived and organized in the Rayyan reference manager.

-Stage 3 - Selection of studies

Rayyan reference manager, two independent reviewers conducted a blind analysis of the titles and abstracts of the 160 studies identified, to define the possible studies eligible for inclusion.

The inclusion criteria were defined as follows: articles available in full; published in Portuguese or English; surface-only protocols or surface treatment; union test used: “microtensile”, “tensile”, “microshear” or “shear”.

The exclusion criteria were: cementation protocols without the presence of ceramic surface treatment; studies with other types of ceramics; other literature reviews; preliminary reports. At the end of this stage, 50 articles were selected for full text reading.

-Step 4 - Data mapping

The 50 works were read in full to select studies eligible for inclusion. The mapped data was entered into a form using the Excel database program.

**Figure 1:**
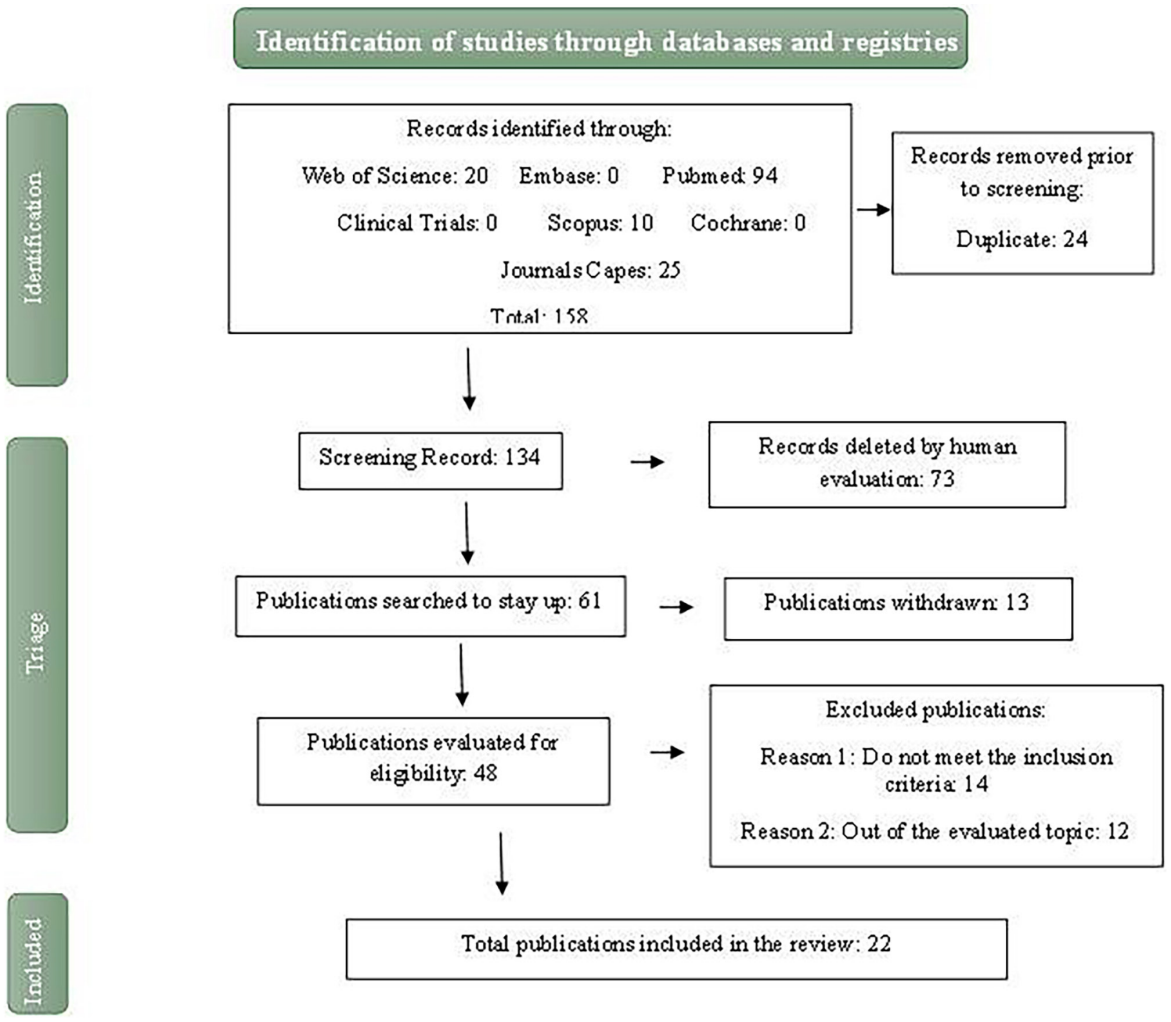
PRISMA Flow Diagram.

To define the studies included in this work, the data extracted from the complete reading of the articles were: authors; year of publication; kind of study; goals; material used; surface treatment; bonding agent; type of substrate; group control; experimental group; aging; adhesive strength test; results; manufacturer’s protocol; material cost. After reading the studies in full, 24 articles were included in this scoping review (Fig. 1).

-Step 5 - Meeting, summary and reporting of results

It consists of gathering, summarizing and reporting the results, presenting an overview of all the material reviewed. This step corresponds to items 4 and 5 of this work.

## Results

The articles were searched through scientific databases: Web of Science (20 articles), Embase (0 articles), PubMed (96 articles), Clinical Trials (0 articles), Scopus (10 articles), Cochrane (9 articles) and Periódicos da Capes (25 articles) (Fig. 1). With the following descriptors; ‘cementation’ and ‘hybrid ceramic’ and ‘bond strength ‘.

A total of 160 scientific articles were found in the analyzed databases, during the period from 2008 to 2024, with the following inclusion criteria: a) hybrid ceramics, b) surface treatment protocol and c) which bonding test was presented. The exclusion criteria were studies that did not involve hybrid ceramics (Fig. 1).

The articles were placed in the EndNote online software, where duplicates were excluded, reaching a total of 136 possible articles for carrying out the systematic review, thus the remaining articles were allocated to the RayYan platform, where it was possible to organize and analyze all articles in full. After excluding works that did not meet the research inclusion criteria, 24 articles remained that were used for this integrative review ( Table 1, Table 2).

In this work, the following data were extracted from the selected articles: Year of publication, the type of study published, what test was carried out, its results, the type of material and its adhesion system used together with the surface treatment carried out on the prosthetic part. Data presented by the authors themselves, whether in Tables or in the text ( Table 3, Table 4).

We can highlight the most used ceramics on the market and in the research used in this study, such as:

• Lava Ultimate (3M ESPE) – nanoceramic resin (80% - zirconia and silica and 20% - polymer); Bonding agent: Silane or universal Single bond

• Vita Enamic (VITA) - hybrid ceramic infiltrated by polymer (86%- ceramics and 14%- polymer); Bonding agent: HF 5% for 60s + silane

• Mazic Duro (Vericom) - nanoceramic resin (80% - zirconia and silica and 20% - polymer); Bonding agent: Alumina blasting + HF 20s + silane

• Cerasmat (GC) - flexible hybrid ceramic (71%- ceramic 29%- polymer); Bonding agent: Alumina + silane blasting

• Shofu block HC (Shofu - nanoceramic resin (61% ceramic and 39% - polymer); Bonding agent: Blasting with 10s alumina + cleaning the blasted surface with alcohol and drying in air free of water and oil + silane

• Katana Avencia (Kuraray) - nanoceramic resin (62% ceramic and 38% - polymer); Bonding agent: Alumina + silane blasting

• Crystal Ultra (DigitalDental) - nanoceramic resin (70% ceramic and 30% - polymer); Bonding agent: Protocol not specified by the manufacturer

We can observe that they all have a greater amount of ceramic in their composition in relation to their polymeric portion.

## Discussion

This integrative review sought to evaluate the different existing protocols for cementing hybrid ceramics, showing which materials are most efficient for treating their surface, whether mechanically or chemically, creating adequate roughness in the microstructure of the ceramic, thus promoting better adhesion results. to the cement used.

As it is a relatively recent material in dentistry, there is still no consensus on the ideal methodology for the most favorable surface treatment to strengthen the union between resin cement and hybrid ceramics to be followed, being modified depending on the material used, the manufacturer and the physical/chemical characteristics of each material (7).

The use of silane as a bonding agent between hybrid ceramics and resin cement is important in several studies presented in Table (8), the Tribst study (9) shows that for a durable bond strength with silane, we must perform 20s of active application followed by 40 seconds of waiting time, which is enough to not require the use of HF. Another work that does not use HF as a surface treatment is the Tekce study (10), which shows the application of alumina blasting, a promising treatment for hybrid ceramics, being used for 30 seconds on its surface, creating the desired roughness for joining to the surface. cement, however, it warns that prolonged use of blasting reduces the values of microtensile bond strength. To evaluate the influence of HF as a surface treatment on hybrid ceramics, the study of Dmonmez (11) shows us tests carried out at different concentrations of HF (5% and 10%) at different exposure times of the product (30s, 60s and 90s), thus we observe that we were able to obtain the appropriate change in the surface using the product at a concentration of 5% with 30s in contact with the ceramic being the most appropriate treatment option for this type of material, and can be considered the gold standard treatment protocol for hybrid ceramics, works by other authors such as Motevasselian (4), Campos (8) and El-Damanhoury (12) share the same ideas showing that HF can improve bond strength to resin cement. On the other hand, Avram (13) show that from a statistical point of view, the conditioning time with HF and its concentration do not affect the change in the microstructure of the hybrid ceramic, and can be used in different ways and still obtain the results desired by HF.

In studies by Barutcigil (14), they show that the absence of some type of surface treatment drastically reduces the bond strength of hybrid ceramics to resin cement, and changing the microstructure of the ceramic, whether with HF, sand blasting or alumina, should always be used. which aims for good results in the union of materials and reinforces that the use of HF 5% for 60s is the most suiTable for adhesion of the characterization layer to the hybrid ceramic.

Regarding the microtensile bond strength in the repair of a hybrid ceramic using composite resin, the authors Bayazit (2) and Fathy (15) show that it is extremely necessary to pre-treat the ceramic before applying the universal adhesive, and the use of HF and silane may be the best strategies to optimize bond strength.

Hybrid ceramics, when pre-treated as recommended by their manufacturers, show encouraging adhesion performance for adhesive cementation, as shown by studies by Frankenberger (3), where they show us that when selecting their recommended surface treatment together with the most suitable bonding agent for each material, it will increase bond strength leading to the desired clinical success. Thus, it was seen that Vita ENAMIC presented greater bond strength when compared to Lava Ultimate in the protocols recommended by the manufacturers, however in the Penumans (8) article, the use of HF, silane or HF+silane as surface treatment in Lava Ultimate, were obtained the highest bond bonds when compared to Vita Enamic.

The possible use of Er:YAG laser as a form of surface treatment in hybrid ceramics is highlighted in studies by Celik16 and El-Damanhoury (12), where they point out its questionable use in hybrid ceramics, as its use can cause a layer damaged by heat, which may be poorly adhered to the internal surface of the substrate, leading to fragmentation of the ceramic crystals, weakening the bond to the resin cement and evidence that chemical corrosion carried out by HF 5% for 60s is the most effective surface treatment for hybrid ceramics.

Other selected studies have obtained significant responses: in studies by Peumans (17), surface mechanical treatment significantly influenced the bond strength of Lava Ultimate; for Awad (18), the application of silane-based primer increased tensile strength after 24 h and after thermocycling compared to the universal silane-containing adhesive and the universal silane-free adhesive; according to Castro (19), Cerasmart had the lowest modulus of elasticity averages and the highest flexural strength averages in relation to Vita Enamic; Dhesi’s studies (20) point out that when ceramics are bonded to titanium bases, the type of ceramic and cement influences the bond strengths, along with the substrate protocols. As for the material, Beyabanaki (21) shows that Crystal Ultra showed higher adhesive strength in relation to the materials studied. According to Jassim (22), conventional treatment increased surface roughness compared to plasma treatment for all materials, and the contact angle decreased after plasma treatment. Finally, Dogan’s study (23) shows that TBC surface treatment produced better repair results for aged hybrid ceramics and additional silane application did not increase the significant values for the groups tested. Nafisifard (24), on the other hand, evaluated that the addition of 4 META at all concentrations significantly improved the μTBS to the enamic ceramic in the use of double-curing and photocurable cement.

## Conclusions

Despite the different surface treatment protocols for hybrid ceramics existing in the scientific literature, HF corrosion remains the gold standard for the treatment of hybrid ceramics, improving considerably the adhesion strength, its absence drastically reduced the microshear bond strength between the ceramic and the bonding agent. Etching with 5% hydrofluoric acid for 60 s is the most suiTable treatment for adhesion of the characterization layer to the hybrid ceramic.

## Figures and Tables

**Table:** General data of the studies included in the review (Part 1).

Author (Year)	Study characteristics	Treatment Protocol (s) of surface Studied
Elsaka (1) (2014)	Objective: Evaluate the effect of different treatments surfaces in microtensile adhesive strength of new CAD/CAM restorative materials for self-adhesive resin cement. Ceramic System: Lava Ultimate and Vita Enamic Substrate: Composite resin Union Agent: Self-Adhesive, Resin Cement (Bifix SE, YOU GmbH) Rehearsal: Microtraction (µ-TBS) Aging: Thermal (Distilled water 37ºC for 30 days)	1.Without Treatment; 2. Silane (Ultradent);; 3.Jat. Al _2 _O _3 _(110µm); 4.Jat. Al _2 _O _3_ (110µm) + Silane; 5.HF9%; 6.HF9%+ Silane
Frankenberger et al. (3) (2015)	Objective: Evaluate the adhesive bonding performance of recently introduced tooth-colored CAD/CAM materials after different pretreatment protocols and using different cementation materials. Ceramic System: Lava Ultimate and Vita Enamic Substrate: --- Union Agent: 1.Adhesive Resin Cement (Calibra, Dentsply) with self-conditiong adhesive; 2.Self Adhesive Resin Cement (RelyX Unicem, 3M ESPE) Rehearsal: Microtraction (µ-TBS) Aging: Thermocycling (10,000 cycles 5ºC/55º)	1.Without Treatment; 2.Silane Silane (does not specify); 3.Jat. Al_2_O_3_(50µm); 4.Jat. Al _2 _O _3_ (50 µm) + Silane; 5.HF5%; 6.HF 5% + Silane
Bottino et al. (7) (2015)	Objective: To evaluate the adaptation of feldspathic and polymer-infiltrated ceramic inlays (CAD/CAM) and the bond strength to dentin after adhesive cementation. Ceramic System: Vita Enamic Substrate: Human Teeth Bonding Union Agent: Adhesive Resin Cement (Panavia F2.0 Kuraray Medical Inc.) associated with ED Primer (Kuraray Medical Inc) Rehearsal: Microtraction (µ-TBS) Aging: Thermomechanical (2,000,000 cycles, load 200 N, 3.8 Hz (5°C, 37°C-55° C)	1. HF10%+ Silane (Monobond S)
Peumans et al. (17) (2016)	Objective: Evaluate the influence of different surface treatments of CAD/CAM materials on the bond strength of two cementing composites. Ceramic System: Lava Ultimate and Vita Enamic Substrate: Blocks of the studied ceramic system Union Agent: Adhesive Resin Cements: 1.Clearfil Esthetic 2.Panavia SA Both with Heliobond adhesive (Kuraray Medical Inc.) Rehearsal: Microtraction (µ-TBS) Aging: Thermal (Distilled water at 37ºC for 7 days	Mechanic: 1.Without Treatment; 2.Jat. Al _2 _O _3 _ (50µm); 3.Jat. Al _2 _O _3 _ (50µm) + silica particles (3M CoJet ESPE); Chemical: 1.HF5%; 2.Silane (Monobond Plus); 3.HF5% + Silane
Campos et al. (8) (2016)	Objective: Evaluate the effect of different treatments surfaces in resistance to microtraction between resin cement and a hybrid ceramic. Ceramic System: Vita Enamic Substrate: Composite resin Union Agent: Adhesive Resin Cement Panavia SA (Kuraray Medical Inc) Rehearsal: Microtraction (µ-TBS) Aging: Thermocycling (6,000 cycles /5°C/55°C) + 60 days of immersion (distilled water 37°C)	1.Without Treatment; 2. Phosphoric acid 37% + Silane (Clearfil - Primer and Activator) 3. HF10% +Silane; 4. Jat. Al _2 _O _3 _ (30µm) + silica particles (CoJet)
Tekçe et al. (10) (2018)	Objective: Evaluate the effect of blasting prolonged resistance to adhesive resin cement bond dual in hybrid CAD/CAM ceramics. Ceramic System: Lava Ultimate and Vita Enamic Substrate: Blocks of the studied ceramic system Union Agent: Self-Adhesive Resin Cement RelyX Ultimate (3M ESPE) Rehearsal: Microtraction (µ-TBS) Aging: Thermocycling (5,000 cycles / 5°C/55°C)	1. Jat. Al _2 _O _3 _ /15 sec. + Silane; 2. Jat. Al _2 _O_3 _/ 30 sec.+ Silane; 3. Jat. Al_2 _O_3 _/ 60 sec + Silane * Al _2 _O _3 _ (50µm)*Silane = Clearfil (Primer and Activator)
Çelik et al. (16) (2018)	Objective: Evaluate the effects of different treatments surfaces in resistance to shear of a cement resinous bonded to various ceramic resin matrices. Ceramic System: Lava Ultimate, CeraSmart and Vita Enamic Substrate: Composite resin Union Agent: --- Rehearsal: Shear (SBS) Aging: Thermocycling (5,000 cycles / 5°C/55°C)	1.Without Treatment; 2.Jat. Al2O3 (50µm) + silica particles (CoJet 3M ESPE) + Primer + Sticker Universal; 3.Nd:YAG laser + Primer + Sticker Universal *Adhesive Universal = Single Bond Universal
Bayazıt (2) (2019)	Objective: To evaluate the effects of different combinations of self-adhesive resin cements and surface treatments on the microtensile adhesive strength of different CAD/CAM resin matrix ceramics. Ceramic System: Lava Ultimate and Vita Enamic Substrate: --- Union Agent: Self-Adhesive Resin Cement 1.RelyX U200 (3M ESPE); 2.SET PP (SDI Dental Ltd) Rehearsal: Microtraction (µ-TBS) Aging: ---	1.Without Treatment; 2.HF9.5%+ Sticker Universal; 3.Jat. Al _2 _O _3 _ (50µm) + Adhesive Universal * Silano = Single Universal Bond
Motevasselian et al. (4) (2019)	Objective: Evaluate the effect of different surface treatments of a hybrid ceramic, Vita Enamic, on the microtensile bond strength to resin cement. Ceramic System: Vita Enamic Substrate: Blocks of the studied ceramic system Union Agent: Adhesive Resin Cement Duo-link Universal (Bisco Inc.) Rehearsal: Microtraction (µ-TBS) Aging: ---	1.Phosphoric acid 35%; 2.Jat. Al2O3 (50µm) + Silane (BisSline Bisco); 3.HF9.5%+ Silane; 4.Laser 2W/Er:YAG; 5.Laser 3W/Er:YAG
Barutcigil et al. (14) (2019)	Objective: To evaluate the effects of various surface treatment methods on the shear strength of self-adhesive resin cement to a new CAD/CAM hybrid ceramic material. Ceramic System: Vita Enamic Substrate: --- Union Agent: Self-Adhesive Resin Cement RelyX U200 (3M ESPE) Rehearsal: Shear (SBS) Aging: ---	1.Without Treatment; 2.Jat. Al _2 _O _3 _ (30µm) + silica particles (CoJet EM ESPE); 3.Jat. Al _2 _O _3 _ (50µm); 4.HF10%; 5.Sticker Universal; 6.Laser 2W/ Er,Cr:YSGG *Silano = Single Universal Bond
Awad et al. (18) (2019)	Objective: To evaluate the effect of universal adhesives and silane on the microtensile adhesive strength of resin cement to a Vita Enamic hybrid ceramic. Ceramic System: Vita Enamic Substrate: Blocks of the studied ceramic system Union Agent: Adhesive Resin Cement Variolink Aesthetic DC (Ivoclar Vivadent) Rehearsal: Microtraction (µ-TBS) Aging: Thermocycling (5,000 cycles / 5°C/55°C)	1.HF4.6%+ Silane (Pulpdent); 2.Sticker Universal /Clearfil Primer and Activator; 3.Sticker Universal/TetricNBond Universal
Castro et al. (19) (2020)	Objective: To evaluate the effect of 1 year of water storage and surface treatments on the shear strength of two mixed cements adhered to CAD/CAM resin matrix ceramics (CMRs) and mechanical properties of CMRs. Ceramic System: Lava Ultimate, Vita Enamic, CeraSmart and ILC Epricord Substrate: --- Union Agent: 1Cimento Resinoso Adesivo Panavia V5 (Kuraray Noritake Dental) 2. Cimento Resinoso Autoadesivo RelyX Ultimate (3M ESPE) Rehearsal: Shear Aging: Thermal (Distilled water at 37ºC for 1 year)	Group A / Panavia 1. Manufacturer's Protocol; 2. Plasma; 3. Plasma + Silane (Clearfil for Lava Ultimate and Vita Enamic; Ceramic Primer II GC for CeraSmart) Group B / RelyX Ultimate 1. Manufacturer's Protocol; 2. Plasma; 3. Plasma + Silane (Scotchbond for Lava Ultimate and Vita Enamic; Ceramic Primer II GC for CeraSmart)

**Table 2:** General data of the studies included in the review (Part 2).

Author (Year)	Study characteristics	Treatment Protocol (s) of surface Studied
Donmez et al. (1) (2020)	Objective: To evaluate the influence of different concentrations and durations of hydrofluoric acid (HF) and Monobond Etch & Prime (MEP) conditioning on the surface roughness (Rum) of different CAD/CAM materials and the shear strength (SBS) between these materials at a self-adhesive resin cement. Ceramic System: Vita Enamic Substrate: --- Union Agent: Self-Adhesive Resin Cement RelyX U200 (3M ESPE) Rehearsal: Shear (SBS) Aging: Thermocycling (5,000 cycles / 5°C/55°C)	1.No treatment; 2.Self-Etching Primer 60 sec.; 3.Self-Etching Primer 120 sec.; 4.HF5% 60 sec + Silane (Monobond Plus / Ivoclar Vivadent); 5.HF5% 120 sec + Silane; 6.HF9.5% 60 sec. + Silane; 7. HF9.5% 120 sec. + Silane *Primer Self-etching = Monobond Etch & Prime / Ivoclar Vivadent.
Ustun & Ayaz (6) (2021)	Objective: Evaluate the effect of 3 different cementing systems after thermal aging on the shear strength of different CAD/CAM materials. Ceramic System: Vita Enamic and CeraSmart Substrate: Human tooth Union Agent: Self-Adhesive Resin Cement RelyX Ultimate (3M ESPE); 2. RelyX U200 (3M ESPE) Rehearsal: Shear (SBS) Aging: Thermocycling (5,000 cycles / 5°C/55°C)	1. HF5% + Silane (Ultradent Silane Porcelain) + Sticker Universal (3M ESPE)
Dhesi et al. (20) (2021)	Objective: Evaluate the effect of cementing titanium infrastructures to different types of ceramics and hybrid materials, with a variety of cements, as well as the effect of adhesive agents. Ceramic System: Lava Ultimate and Vita Enamic Substrate: Titanium Union Agent: Self-Adhesive Resin Cement 1.Multilink hybrid abutment (Ivoclar Vivadent); 2.TheraCem (Bisco) Adhesive Resin Cement Panavia V5 (Kuraray Noritake Dental) Rehearsal: Shear (SBS) Aging: Thermocycling (5,000 cycles / 5°C/55°C)	1.HF4.5%+ Neutralizing + Silane (Monobond Plus /Ivoclar Vivadent) Ceramics Etching Gel IPS Ceramics Neutralizing Powder / Ivoclar Vivadent
El-Damanhoury et al. (12) (2021)	Objective: To evaluate the effect of laser on roughness, surface topography and bond strength to resin cement compared to other chemical and microabrasion pretreatments of different computer-aided manufacturing materials. Ceramic System: Lava Ultimate, Vita Enamic, CeraSmart and Shofu Block HC Substrate: Composite resin Union Agent: Self-Adhesive Resin Cement MultilinkN (Ivoclar Vivadent) Rehearsal: Shear (SBS) Aging: Thermocycling (5,000 cycles / 5°C/55°C)	1.Silane; 2;Er:YAG; 3.HF4.5%+ Silane; 4.Silane; 5.Jat. Al _2 _O _3_ (50µm) + Silane *Silane = Monobond Plus
Tribist et al. (9) (2021)	Objective: To evaluate the effect of active application of a self-etching primer on the bond strength of different dental CAD/CAM materials. Ceramic System: Vita Enamic Substrate: --- Union Agent: Adhesive Resin Cement VariolinkN (Ivoclar Vivadent) Rehearsal: Shear (SBS) Aging: Thermocycling (5,000 cycles / 5°C/55°C)	1.Primer Self conditioning 20 sec (active application) + Action Time 40 sec.; 2.Primer Self-conditioning and 60 sec (passive application) * Primer Self-etching = Monobond Etch & Prime / Ivoclar Vivadent,
Beyabanaki et al. (21) (2022)	Objective: To evaluate the bond strength of three monolithic hybrid nanoceramics/resins and a lithium silicate reinforced with zirconia to resin cement after thermocycling. Ceramic System: Ultimate and Enamic Ultra Substrate: --- Union Agent: Adhesive Resin Cement Panavia F2 (Kuraray Noritake Rehearsal: Micro shear (µSBS) Aging: Thermocycling (2,000 cycles /5°C/55°C)	Lava Ultimate and Crystal Ultra: 1. Jat. Al _2 _O _3_ (50µm) + Primer Ceramic Vita Enamic: 1. HF10%+ Ceramic Primer (Clearfil Ceramic Primer Plus/ Kuraray)
Avram et al. (13) (2022)	Objective: To evaluate the influence of hydrofluoric acid (HF) and conditioning time on microshear strength (µSBS) between double-curing resin cement and glass-ceramic materials, leucite-reinforced ceramics and hybrid ceramics. Ceramic System: Vita Enamic Substrate: --- Union Agent: Self-Adhesive Resin Cement RelyX Ultimate (3M ESPE) Rehearsal: Micro shear (µSBS) Aging: ---	1. HF9.5% 30 sec.+ Universal Adhesive Single Bond Universal (3M ESPE); 2. HF9.5% 60 sec. + Universal Adhesive; 3. HF9.5% 90 sec. + Sticker Universal
Fathy et al. (15) (2022)	Objective: To evaluate the effect of different surface treatment methods on the microtensile strength (µTBS) of two different computer-aided hybrid ceramics (CAD/CAM). Ceramic System: Lava Ultimate and Vita Enamic Substrate: Human tooth Union Agent: Self-Adhesive Resin Cement RelyX U200 (3M ESPE) Rehearsal: Microtraction (µTBS) Aging: ---	1.Without Treatment; 2.Jat. Al _2 _O _3_; 2.(50µm) + Adhesive Universal (Single Bond Universal (3M ESPE); 3.Jat. Al _2 _O _3_ (50µm) + Silane (BisSilane Bisco); 4.HF9.5% Sticker Universal; 5. HF9.5% Silane
Jassim & Majeed (22) (2023)	Objective: Evaluate and compare the effect of plasma treatment versus conventional treatment on microshear strength (µSBS), surface roughness and wettability of three different CAD/CAM materials. Ceramic System: CeraSmart Substrate: --- Union Agent: Self-Adhesive Resin Cement G-CEM One, (GC Corporate) Rehearsal: Micro shear (µSBS) Aging: ---	1.Primer Universal (GMulti Primer (GC corporation); 2.HF < 5% + Universal Primer; 3.HF < 5% + Plasma + Primer Universal
Piemjai & Donpinprai (5) (2023)	Objective: To evaluate the tensile strength (TBS) of dental veneers manufactured from experimental biopolymers and commercial hybrid materials bonded to enamel using two different cementing adhesives. Ceramic System: Vita Enamic, Shofu Block HC and Katana Avencia Substrate: bovine tooth Union Agent: Self-Adhesive Resin Cement 1.SuperBond B&C (Sun Medical); 2.RelyX U200 (3M ESPE) Rehearsal: Traction (TBS) Aging: ---	1. HF5%+ Shofu Block HC and Katana Avencia Ceramic Primer 2. Jet. Al _2 _O _3 _ (50µm) + Universal Ceramic Primer Adhesive = Universal Ceramic Primer C (Sun Medical) when using SuperBond B&C resin cement C (Sun Medical) RelyX Ceramic Primer (3M ESPE) when using RelyX U200 resin cement (3M ESPE)
Dogan & Karaman (23) (2024)	Objective: Evaluated different surface treatment methods and different universal adhesives with or without silane and the bonding repair strength of CAD/CAM hybrid ceramic restorations. Ceramic System: CeraSmart Substrate: Composite resin Union Agent: Single Bond Universal 3M Espe Futurabond M+ VOCO Clearfil Universal Bond Quick Kuraray Tokuyama Universal Bond Tokuyama Dental Rehearsal: Shear (SBS) Aging: Thermocycling (5,000 cycles / 5°C/55°C)	1. Without Treatment; 2.Diamond bur (DB); 3.Hydrofluoric acid (HF); 4.Tribochemical silica coating (TBC)
Nafisifard et al. (24) (2024)	Objective: It evaluated the effect of the addition of 4 META at concentrations of 0%, 2.5%, 5% and 10% to silane on the adhesive strength to microtensile strength of light-cured and double-cured resin cement to ceramics. Ceramic System: Vita Enamic and Celtra Duo Substrate: --- Union Agent: 1. Amber APS (FGM, Joinville, SC, Brazil) bonding agent; 2. Allcem Veneer lightcure cement Rehearsal: Shear Aging: Thermocycling (2,500 cycles / 5°C/55°C)	1. Ácido HF a 5% (30 s para Celtra Duo e 60 s para blocos Enamic); 2. Silano com 4 META (0%) -1 min e seco com spray de ar por 15 s.

**Table 3:** Main results found in each study (Part 1).

Author (Year)	Main results	Effectiveness of the Protocol
Elsaka (1) (2014)	1. Surface treatment, type of CAD/CAM restorative material, and water storage periods showed a significant effect on μTBS; 2. For the Vita Enamic system, the surface treatment with HF9% + S showed higher adhesive strength values ​​compared to the other treatments; 3. For Lava ultimate there was no significant difference.	1.Manufacturer's protocol used; 2. The manufacturer's protocol, for the two materials analyzed, presented higher adhesion values ​​compared to the other surface treatments carried out.
Frankenberger et al. (3) (2015)	1. LAVA ULTIMATE with cementing adhesive: groups G3/Blasting and G4/Blasting + Silane showed the highest values ​​of adhesive strength; with self-adhesive cementation: groups G3/Blasting and G4/Blasting + Silane showed the highest values ​​of adhesive strength; 2. VITA ENAMIC with cementing adhesive: groups G5/HF5% and G6/HF5% + Silane showed the highest values ​​of adhesive strength; with self-adhesive cementation: the G6/HF5% + Silane group presented the highest values ​​of adhesive strength.	1. Manufacturer's Protocol Used; 2. The manufacturer's protocol, for Vita Enamic, showed higher adhesion values ​​compared to other surface treatments carried out; 3. The use of Jat. Al2O3 (50µm), for Lava Ultimate, showed higher binding values.
Bottino et al. (7) (2015)	1. Group G2/Vita Enamic presented lower bond strength values ​​compared to group G1/VitaBlock Mark II; 2.The contact angle of the polymer-infiltrated ceramic was also greater.	1. Manufacturer's Protocol Used; 2. The manufacturer's protocol showed excellent adherence values.
Peumans et al. (17) (2016)	1. The adhesion of the six CAD/CAM materials was significantly influenced by the surface treatment; 2. Mechanical surface treatment significantly influenced the bond strength of Lava Ultimate.	1.Manufacturer's protocol used; 2. The manufacturer's protocol, for both materials, presented higher adhesion values ​​than other surface treatments.
Campos et al. (8) (2016)	1. The affected surface treatment significantly improves the bond strength before and after aging; 2. Before aging, the samples from the blasting groups with aluminum oxide and silica particles, HF10% and 35% phosphoric acid obtained the highest bond strength values.	1.Manufacturer's protocol used; 2. The manufacturer's protocol showed higher adhesion values ​​compared to other surface treatments.
Tekçe et al. (10) (2018)	1. At baseline, group 1, 2, and 3 exhibited statistically similar µTBS results for LAVA; 2. After 5,000 thermocycles, µTBS values ​​decreased significantly for each block.	1. Did not use the Manufacturer's Protocol; 2. The use of Jat. Al _2 _O _3 _/15 sec. + Silane, presented higher values.
Çelik et al. (16)(2018)	1. Blasting and Laser significantly increased the SBS values ​​of CeraSmart (G8 and G9) but this did not happen for all materials, as the highest shear values ​​were observed for the Vita Enamic samples treated with blasting (G4) and for the samples Lava Ultimate when treated with laser (G7).	1. Did not use the Manufacturer's Protocol; 2. The use of Jat. Al _2 _O _3 _ (50µm) + silica particles (CoJet 3M ESPE) + Silane + Adhesive, showed higher binding values; 2. The manufacturer's protocol would indicate higher values.
Bayazıt (2) (2019)	1.No statistically significant difference was observed between the control groups; 2. Lava Ultimate when treated with HF9.5% + Universal Adhesive, cemented with RelyX U200 showed the highest microtraction values; 3. The Vita Enamic group, blasted with Al _2 _O _3 _ + Universal Adhesive and cemented with RelyX U200, showed the highest microtraction values ​​compared to the group treated with HF9.5% + Universal Adhesive, cemented with RelyX U200 or SET PP; 4. Surface treatment, regardless of the type, had a greater effect on microtensile values ​​compared to the effect of the ceramic matrix and cement used.	1. Did not use the Manufacturer's Protocol; 2. The manufacturer's protocol showed higher adhesion values ​​compared to other surface treatments carried out by the author.
Motevasselian et al. (4) (2019)	1. µ-TBS was influenced by surface treatment methods and thermocycling significantly decreased bond strength values ​​in all groups;	1. Manufacturer's Protocol Used; 2. The manufacturer's protocol showed higher adhesion values ​​in relation to the surface treatments carried out.
Barutcigil et al. (14) (2019)	1. The group treated with universal adhesive showed the highest bond strength values, being statistically different from the control group.	1. Did not use the Manufacturer's Protocol; 2. The manufacturer's protocol showed higher adhesion values ​​compared to the surface treatments carried out by the author.
Awad MM. et al. (18) (2019)	1. Application of silane-based primer resulted in higher tensile strength after 24 hours and after thermocycling compared to universal adhesive with and without silane; 2. The TBS values ​​of all groups were significantly reduced after thermocycling; 3. There was a significant difference between the tensile values ​​of the universal adhesive with silane and the universal adhesive without silane after 24 h; 4. The silane-free universal adhesive showed significantly higher tensile strength after thermocycling; 5. Adhesive failure was the most common in all groups. 6. There were superficial topographic changes after hydrofluoric acid.	1.Used Manufacturer's Protocol; 2. The use of the manufacturer's protocol showed greater bond values compared to other surface treatments carried out.

**Table 4:** Main results found in each study (Part 2).

Author (Year)	Main results	Effectiveness of the Protocol
Castro et al. (19) (2020)	1. The groups treated according to the manufacturer exhibited higher SBS than the plasma and plasma + binding agent groups for all indirect materials, cements and storage periods tested; 2. Overall, the RelyX Ultimate had a higher average SBS than the Panavia V5; 3. After 1 year of storage in water, all groups showed a significant reduction in SIC, except for some groups that followed the manufacturer's instructions; 4. ILC presented the lowest values ​​of elastic modulus and flexural strength; 5. Cerasmart had the lowest average modulus of elasticity and the highest average flexural strength, and Vita Enamic had the highest average modulus of elasticity and lowest flexural strength for both storage periods.	- 1. Manufacturer's Protocol Used; 2. The manufacturer's protocol showed higher adhesion values ​​compared to other surface treatments.
Donmez et al. (11) (2020)	1.Monobond Etch & Prime may be a preferable method to achieve high bond strength values.	———-
Ustun & Ayaz. et al. (6) (2021)	1. The highest values ​​of bond strength were found in Vita Suprinity with prior acid treatment without thermal aging and the lowest strength was with Vita Enamic with prior acid conditioning; 2. Furthermore, thermal aging significantly reduced the bond strength values ​​of all ceramic materials, regardless of the cementing procedure.	1. Did not use the Manufacturer's Protocol; 2. The manufacturer's protocol showed higher bond values ​​compared to surface treatments.
Dhesi et al. (20) (2020)	- 1. Ceramic and cement type significantly affected shear strength, while thermocycling did not; 2. When ceramics are bonded to titanium bases, the type of ceramic and cement have an impact on bond strengths, along with the conditioning and adhesion protocols for each substrate; 3. Equal affinity of the tested cements was found for ceramic, hybrid and titanium materials.	1. Manufacturer's protocol used; The manufacturer's protocol showed excellent adherence values.
El Damanhoury et al. (12) (2021)	- 1. Etching IPS e.max CAD lithium disilicate and Vita ENAMIC PICN with hydrofluoric acid or self-adhesive ceramic primer resulted in the highest shear values; 2. Lava ultimate, Shofu and Vita Enamic had similar values ​​when treated with 3 W, 6 W and alumina blasting. 3; The highest surface roughness values ​​were for the Lava Ultimate, Cerasmart and Vita Enamic groups when treated with 6 W, while the lowest shear values ​​were obtained for Cerasmart when treated with self-adhesive ceramic primer and IPS e.max CAD lithium disilicate when treated with self-adhesive ceramic primer or 3 W laser; 4. Only Shofu and Cerasmart indicated a significant correlation between surface roughness and adhesion.	1.Did not use the Manufacturer's Protocol; 2. The manufacturer's protocol showed excellent adherence values.
Tribist et al. (9) (2021)	- 1. The aging process has a negative impact on the bond strength for all groups, except for Lithium Disilicate; 2. Zirconia reinforced with lithium silicate and leucite showed high bond strength values ​​for primer application; however, after aging, the bond strength value was reduced; 3. The hybrid ceramic showed reduced bond strength values ​​regardless of primer application.	1. Did not use the Manufacturer's Protocol; 2. The use of Silane (20 seconds of active application + 40 seconds of Action Time) showed excellent adhesion values.
Beyabanaki et al. (21) (2022)	- 1. The differences between the groups were significant and Crystal Ultra showed greater bond strength compared to the three materials.	1. Did not use the Manufacturer's Protocol; 2. The manufacturer's protocol showed excellent adherence values.
Avram et al. (13)(2022)	1. The mean difference in µSBS for leucite-reinforced ceramics was statistically significant (p < 0.05); 2. However, the µSBS values ​​for hybrid ceramics and lithium disilicate ceramics were not affected, from a statistical point of view, by the etching time (p > 0.05).	1. Manufacturer's protocol used; 2. The manufacturer's protocol showed excellent adherence values.
Fathy et al. (15) (2022)	1. Surface treatments significantly increased the µTBS of materials compared to the control group; 2. Cohesive failure of the CAD/CAM restorative material was the most predominant failure mode in all groups, while adhesive failure at the restoration-cement interface was the most predominant failure mode in the group 3 without surface treatment.	1. Manufacturer's protocol used; 2. The manufacturer's protocol showed excellent adherence values.
Jassim & Majeed (22) (2023)	1. For all CAD/CAM materials, the Conventional treatment increased the surface roughness compared with plasma treatment, while the contact angle decreased after plasma treatment.	1. Did not use the Manufacturer's Protocol; The use of HF < 5% + Plasma showed higher union values.
Piemjai & Donpinprai (5) (2023)	1. Experimental results of biopolymer crowns demonstrated the highest average tensile strength with cohesive failure in cementing agents; 2. Adhesive failure at the crown interface was found in other groups; 3. There was no significant difference between the two cementing agents.	1. Manufacturer's protocol used; 2. The manufacturer's protocol showed excellent adherence values; 3. Shofu presented the lowest adhesion values ​​compared to the other materials used.
Dogan & Karaman (23) (2024)	1. The highest SBS values ​​were in samples applied with TUB (p<0.05). The application of silane did not cause a significant difference between the tested groups; 2. Among the applied silane groups, the TUB application caused the highest SBS values. The additional application of silane caused an increase in SBS values ​​for the TUB group and a decrease in values ​​for the CUQ group.	1. Manufacturer's protocol used; 2. Additional silane application did not increase SBS values ​​for almost all groups tested.
Nafisifard et al. (24) (2024)	- 1. The µTBS of Celtra Duo was significantly higher than that of Enamic when bonding to light-cured cement using 4 META; 2. Based on the concentrations of 4 META, there was a significant difference between the Enamic groups with different concentrations of 4 META linked to the dual-curing cement and the light-curing cement.	1.Manufacturer's protocol used; 2. The addition of 4 META (10%) to the silane improved the µTBS of the light-cured cement; 2. The addition of 4 META at all concentrations improved the µTBS of Enamic ceramic with double curing cement.

## Data Availability

The datasets used and/or analyzed during the current study are available from the corresponding author.
